# Special Aspects of Nitrocellulose Molar Mass Determination by Dynamic Light Scattering

**DOI:** 10.3390/polym15020263

**Published:** 2023-01-04

**Authors:** Roman Solovov, Anfisa Kazberova, Boris Ershov

**Affiliations:** Frumkin Institute of Physical Chemistry and Electrochemistry, Russian Academy of Sciences, 117342 Moscow, Russia

**Keywords:** colloxylin, nitrocellulose, hydrodynamic radius, fractionation, dynamic light scattering, polymer

## Abstract

The dynamic light scattering method was successfully applied to determine the molar mass of nitrocellulose. The methodology of nitrocellulose fractionation in acetonic solutions is described in detail; six polymer fractions with monomodal distribution were obtained. It was shown that the unfractionated colloxylin with polymodal molar mass distribution had mass average molecular mass values of 87.3 ± 14.1, 28.3 ± 7.3, and 0.54 ± 0.17 kDa when investigated by the dynamic light scattering method. The viscometric method only provided integral viscosity average molar mass equal to 56.7 ± 5.8 kDa.

## 1. Introduction

Nitrocellulose is a multipurpose, widely used polymer with numerous fields of application [1,2,3,4,5,6,7,8]. It is the main component of smokeless gunpowder [9]. Cellulose nitrates are used as membranes for high-efficiency microporous filters in the pharmaceutical industry [10]. In this regard, it is often used in various biosensors [11,12,13,14,15,16]. Commercial flow immunochromatographic assay kits with nitrocellulose membranes allow the detection of specific biomolecules from the analyte. They provide an accessible platform for prenatal testing, oncogene mutation testing, infectious disease diagnosis, and microbial identification [17,18]. In addition, cellulose nitrates are used to produce composites of cellulose derivatives with soot for cleaning up after oil spills [19,20].

One of the most important parameters of any polymer is the degree of polymerization. Nitrocellulose polymolecularity is due to both by the polymolecularity of the original cellulose and etherification irregularity. This leads to cellulose nitrates with the same average degree of polymerization and with a different degrees of nitration having different physical and mechanical characteristics [21,22,23,24]. As a rule, viscosity average, number average, or mass average degree of polymerization is determined. Finding a convenient and fast method to determine these parameters for nitrocellulose has been the subject of scientific research for many years. It is best to have a universal and convenient method of measuring the molecular mass distribution of a nitrocellulose. These include viscometry, dynamic and static light scattering, and size-exclusion chromatography. Viscometry is the main method for determining the viscosity average molar mass in industry. The method is simple and does not require advanced equipment [25,26,27,28]. The polymerization degree of cellulose nitrates determined by the viscometric method, most commonly, is a mean value of several separate fractions. Separation techniques such as size exclusion chromatography are used to obtain the molar mass distribution [29,30,31]. Size exclusion chromatography results in the separation of molecules according to their hydrodynamic radius. The main limitation of all the above methods is the solubility of nitrocellulose in a desired solvent. New solvents such as ionic liquids have recently been introduced in cellulose chemistry but have not been widely applied in SEC yet [32,33].

Aside from chromatographic methods, certain information about molar mass distribution can be obtained by the molecular fractionation of polymers, i.e., performing a polymer sample fractionation by various methods to receive fractions with different molar masses. The investigation of nitrocellulose molar mass distribution in samples is carried out by traditional fractionation methods, followed by the molar mass determination of each of the fractions, which is a rather complex and time-consuming method. This is complicated by the aging processes of polymer solutions, which create significant problems with results reproducibility [34,35,36].

The literature describes several fractionation methods applicable to cellulose derivatives. Fraction filtration is a method of passing a solution through filters or membranes of various porousness. This method is overly complex and time-consuming, which is further complicated by clogging the filter pores and subsequently increasing pressure [37]. Another fractionation method is fractional dilution, when the entire polymer is placed in a solvent of a certain composition and only a certain fraction of the polymer passes into the solution. However, fractional dissolution is hindered by both the solvent diffusion over the entire volume of the swollen fiber or film and the diffusion of dissolved nitrocellulose chains from the inner layers of the polymer into the solution. Hence, all polymer chains that are hypothetically soluble in a solvent of this composition might not actually dissolve. Therefore, this method often requires repeated fractionation [38].

The preferred method of obtaining fractions with a certain molar mass is the method of fractionation by salting out cellulose nitrates. This method yields fractions limited in volume caused by the necessity of using only low-concentration solutions, but the obtained results are reproducible [38,39]. The further viscometric method of determining molar masses is not absolute. Each “polymer–solvent” system requires a comparison of the results obtained by this method with the data obtained via absolute methods, such as osmometry or light scattering, and further, one should use polymers that have either a narrow or exactly known molecular mass distribution. If the dependence of viscosity on molar mass is not established for a certain “polymer–solvent” system, then the obtained results characterize the average integral value of the molar mass over the entire fraction [35,40].

The light scattering method is one of the main physical methods for studying polymer solutions. This is a reliable and absolute way to determine the molar masses of polymers [41,42,43]. It covers a wide range of molar masses from 10^3^ to 10^8^ Da. These measurements can be taken because polymer macromolecules in solutions exist in swelled coiled form, whose geometric dimensions are in the nanometer range. The size of the coiled polymer is determined by the length of the polymer chain and, accordingly, the molar mass of the polymer. The application of this method is currently not limited to measuring only the molecular parameters of polymers but also includes the determination of other important characteristics, such as the size and shape of macromolecules, polydispersity of the sample, and thermodynamic parameters of intermolecular interaction [44,45,46].

There is static and dynamic light scattering. Static light scattering allows determining the mass average molar mass, the radius of gyration, and the second virial coefficient in solutions. Dynamic light scattering allows us to determine the translational diffusion coefficient and calculate the hydrodynamic radius of polymer chains in solutions [36,47,48,49,50,51]. Static light scattering measurements provide scattering indicatrix—the dependence of the scattering intensity on the measurement angle. The static light scattering method most often is suitable for small molar masses and, accordingly, for small-sized coiled polymers. The dynamic light scattering method is typically used in a wider range of molar masses of polymers. However, the dependencies used in static light scattering are usually implemented to interpret the received scattering intensity data [52,53]. Additionally, before determining the molar mass of the polymer by the dynamic light scattering method, it is required to characterize the viscosity and refractive index of the solvent and polymer solutions by known methods [44].

The dynamic light scattering method with the implementation of dependencies from the static light scattering theory allows expanding the possibilities of the dynamic light scattering method. This work substantiates the possibility of using the dynamic scattering method to obtain the molar mass distribution of nitrocellulose.

The aim of this work is to compare the viscometric method and the modified dynamic light scattering method. For this purpose, monomodal fractions of nitrocellulose from the initial colloxylin are obtained and investigated by these methods. The initial sample of cellulose nitrate is also investigated by these methods.

## 2. Materials and Methods

*Fractionation of the initial commercially available nitrocellulose.* Commercially available low-molar-mass colloxylin was used as the initial stock for obtaining monomodal fractions. To obtain the fractions, a colloxylin solution in acetone was placed on a magnetic stirrer, small portions of water were added until opalescence occurred, and then the solution was stirred for 5 min. After that, a beaker with the opalescent solution was transferred to a thermostat and heated up to 35 °C. After the solution had cleared and all the particles had dissolved, the solution was cooled down in a thermostat at 20 °C and kept at this temperature for 15 min. This operation was carried out in order to eliminate the residual effect of the molar mass distribution data distortion. When the solution cooled down, it was centrifuged (BioSan Laboratory Centrifuge LMC-3000, Biosan, Riga, Latvia) for 15 min at 3000 rpm to separate nitrocellulose precipitation and then decanted; the decantate was fractionated in the same way, and the precipitate was washed with water and dried at 50 °C until it reached constant mass [54].

*Determination of the nitrocellulose nitration degree.* The degree of nitrocellulose nitration was determined by reverse potentiometric titration of nitrogroups in the isolated fractions of nitrocellulose. An aliquot of concentrated sulfuric acid was added to the nitrocellulose solution; the released nitric acid was titrated with a standard Fe(II) solution with a precisely known concentration. Standardization of Mohr’s salt standard solution was carried out by titration with a sodium nitrate solution. All titrations were carried out during cooling in a crystallizer. The end point of titration was established by processing the potentiometric titration curves using Gran’s plot. All experiments were carried out at least 5 times.

*Viscometric determination of molar mass.* The capillary flow time (diameter of the capillary 0.34 mm) of nitrocellulose fractions solutions was determined by the Ubbelohde viscometer (range of viscosity measurements from 0.6 to 3 mm^2^·s^−2^, the maximum deviation of ±0.02 mm, the constant value equal 0.002911 mm^2^·s^−2^). The solution of each of the fractions was diluted, and the viscometer was pre-washed with acetone and dried before measuring each concentration of the solution; the studied nitrocellulose solution was poured into the viscometer and left in the thermostat for 20 min at 20 °C. The flow time of each of the nitrocellulose fractions solutions with different concentrations was measured until ten convergent results were reached. The flow time *t*_0_ of the pure solvent (acetone) was determined similarly. Based on the obtained data, observed specific viscosity of the solutions was calculated using the following formulas:

$$\eta_{sp}=\frac{t}{t_{0}}-1,$$

where *η_sp_* is the observed specific viscosity of the solutions, c.u.; *t* and *t*_0_ are the flow time of the nitrocellulose solutions and pure solvent acetone, respectively, *s*.

After measuring the flow time of the solution, the flow time *t*_0_ of the pure solvent was measured in the say method, having washed the viscometer with it 4–5 times. Then, the dependence *η_sp_ C*^−1^ vs. *C* is plotted, where *C* is nitrocellulose solution concentration in g·100·mL^−1^ and the intrinsic viscosity [*η*]*_sp_* are calculated by extrapolating to zero concentration. The nitrocellulose substitution degree effect on the viscosity of its solution and the correction for the nitrogen content is introduced to compensate for the effect. The intrinsic viscosity of the studied nitrocellulose solution with a nitrogen content of *ω(N)* is reduced to the viscosity of the nitrocellulose solution [*η*]_13.6_ with a nitrogen content of 13.6%, which is accepted as standard in accordance with the following formula [55,56]:

$${\left[\eta \right]}_{13.6}={\left[\eta \right]}_{sp}\cdot {R_{\omega \left(N\right)}}^{13.6}$$

$$lg\left({R_{\omega \left(N\right)}}^{13.6}\right)=\mathrm{lg}\left(1.833-0.0589\cdot \omega \left(N\right)\right)+0.114\cdot \left(13.6-\omega \left(N\right)\right)-0.0137,$$

where *R_ω(N)_*^13.6^ is a conversion factor; *ω(N)* is the nitrogen content in nitrocellulose, %. Then, using the corrected value of the intrinsic viscosity, the average degree of polymerization is calculated using the Newman–Loeb–Conrad formula [54]:

$$P=100\cdot {\left[\eta \right]}_{13.6}.$$

As shown in the works [54,55,56], these formulas can be applied to nitrate cellulose with a nitrogen content of 8 to 14%.

*Determination of molar mass by dynamic light scattering method.* The size of formed micelles was determined on a Delsa Nano C light-scattering instrument (Beckman Coulter, Inc., Brea, CA, USA) at the wavelength of scattered laser radiation λ_0_ = 658 nm. The calculations were carried out using the Delsa Nano Software program package. This method determines the average particle size from the particle size distribution derived from the time correlation functions of scattered light intensity. The hydrodynamic radius was measured at 20 °C.

The hydrodynamic diameter and scattering intensity obtained by the dynamic light scattering method was mathematically processed using the following expressions used in the static light scattering method. The value of the Rayleigh coefficient was calculated using the formula:

$$R\left(\theta \right)=\frac{I\left(\theta \right)\cdot R_{t}}{I_{t}\left(\theta \right)}\cdot \frac{n_{0}}{n_{t}},$$

where *I(θ)* is scattering intensity by the investigated solution at the scattering angle *θ*; *I_t_(θ)* is scattering intensity by a standard solvent (toluene) at the scattering angle *θ*; *R_t_* = 13.40 × 10^−6^ cm^−1^ is the Rayleigh coefficient for toluene [57,58]; *n*_0_ is the refractive index of acetone solvent, *n*_0_ = 1.3589; *n_t_* is the refractive index of toluene, *n_t_* = 1.48899. Then the value of the constant included in the Zimm equation was calculated by using the following formula:

$$K=\frac{4\cdot \pi^{2}\cdot n_{0}^{2}}{N_{A}\cdot \lambda^{4}}\cdot {\left(\frac{dn}{dC}\right)}^{2},$$

where *λ* is the wavelength of scattered laser radiation, *λ* = 658 nm; *N_A_* = 6.023 × 10^23^ mol^−1^ is Avogadro’s number; *n*_0_ is the refractive index of acetone solvent, *n_0_* = 1.3589; *dn/dC* is the concentration increment of the refractive index of a specific fraction of nitrocellulose solution in acetone. The *P(θ)* was introduced to take into account the size and shape parameter of the polymer in solution; *P(θ)* was calculated from the scattering angle and hydrodynamic diameter according to the following formula for figuratively spherical polymer particles in the solution:

$$P\left(\theta \right)=1+\frac{4\cdot \pi^{2}\cdot n_{0}^{2}\cdot sin\left(\frac{\theta }{2}\right)}{\lambda^{2}}\cdot \frac{R_{H}^{2}}{5}$$

where *λ* is the wavelength of scattered laser radiation, *λ* = 658 nm; *n*_0_ is the refractive index of acetone solvent, *n*_0_ = 1.3589; *θ* is the scattering angle, *θ* = 14.6° = 0.2548 radian; *R_H_* is the hydrodynamic radius of nitrocellulose globules in acetone at a certain concentration, nm. The experimentally obtained values, and the parameters calculated from them were processed according to the Rayleigh–Zimm equation:

$$\frac{K\cdot C}{R\left(\theta \right)}=\frac{1}{M_{W}\cdot P\left(\theta \right)}+2\cdot A_{2}\cdot C,$$

where *A*_2_ is the second virial coefficient of the nitrocellulose solution. A calibration curve *K·C·P(θ)·R(θ)^−1^ = f(C·P(θ))* was plotted for each fraction, and the value of 1/*M_W_*, and later *M_W_*, was calculated by extrapolating to the zero value of the *C·P(θ)* product [59,60,61].

*Increment determination of the nitrocellulose fraction solution refractive index.* The increment of the nitrocellulose fraction solution refractive index was determined using the RL-2 refractometer (Poland) equipped with water cooling. The solution of the corresponding fraction was placed in a thermostat for 30 min, then a small part of it was transferred on a refractometer glass equipped with water cooling, kept there at a temperature of 20 °C for 15 min, and then its refractive index was measured. The concentration increment of the refractive coefficients *dn/dC* equal to the slope was determined by the dependence of n^20^ on the C concentration.

*Size exclusion chromatography.* The molar mass of cellulose nitrate was determined by size exclusion chromatography. The size exclusion chromatography measurements were performed in tetrahydrofuran at 30 °C with a flow rate of 1.0 mL min^−1^ using a chromatograph Gilson (Inc., Middleton, WI, USA) equipped with refractive index detector and with two columns PLgel 5 μm MIXED B for the molar mass range 5 × 10^1^–3 × 10^6^ Da. The size exclusion chromatography system was calibrated using narrow dispersed linear poly(methyl methacrylate) standards with molar mass ranging from 8 × 10^1^–2 × 10^5^ Da. A second-order polynomial was used to fit the log*M* versus retention time dependence. The molar mass was recalculated for nitrocellulose using known coefficients of the Mark–Houwink equation [62].

## 3. Results and Discussions

The disadvantage of the dynamic light scattering method is the significant contribution of rotational diffusion, which leads to an increase in the influence of the scattering intensities measurement angle. The best option is to measure the scattering intensities and the hydrodynamic radius at different scattering angles and to plot a Zimm diagram. However, in most cases, the manufacturer does not provide the user with the ability to change the measurement angle and offers only its certain values (at best, two angle options, but usually only one). Thus, a researcher is unable to plot a Zimm diagram. The solution to this problem is to take into consideration the rotational diffusion by introducing a parameter *P(θ)*, which includes the angular momentum based on the hydrodynamic radius.

**Figure 1:**
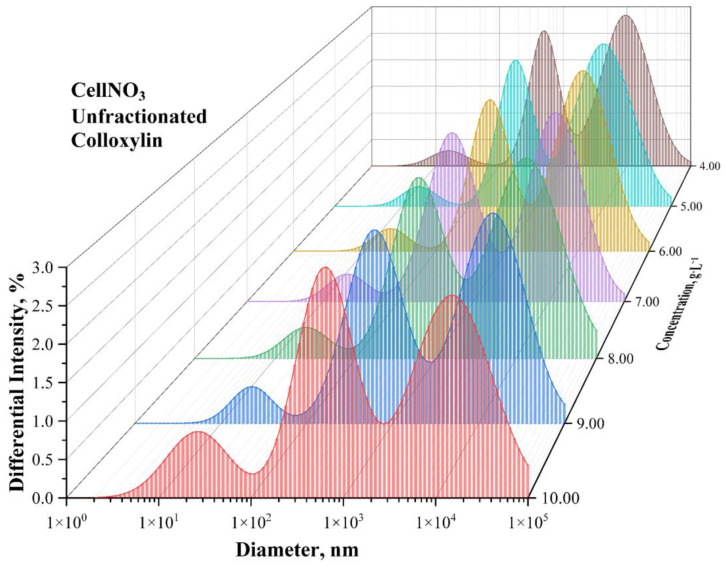
Scattering intensity distribution histograms vs. the hydrodynamic diameter for different concentration (different color) solutions of a unfractionated colloxylin. The data are obtained by the dynamic light scattering method.

Figure 1 shows histograms of the scattering intensity distribution by the size of nitrocellulose globules for the initial sample solution of unfractionated colloxylin in acetone. These distributions are polymodal and cannot be used for correlation comparison of viscometric data and data obtained by the dynamic light scattering method.

**Figure 2:**
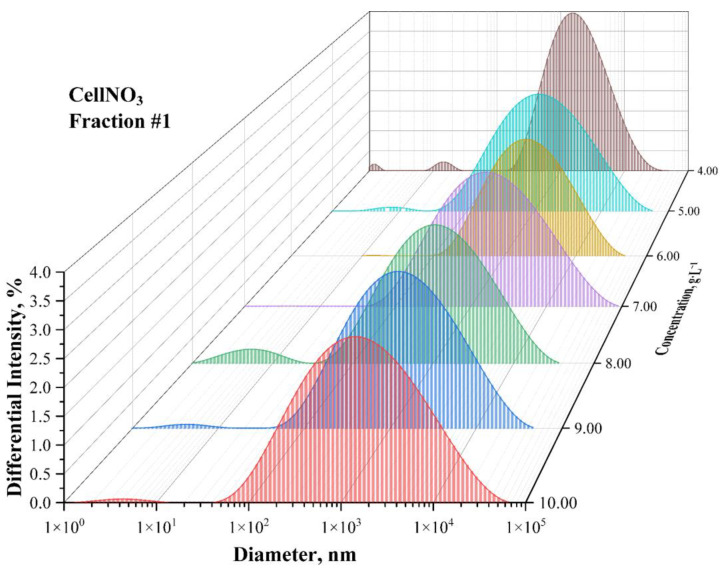
Scattering intensity distribution histograms vs. the hydrodynamic diameter for different concentration (different color) solutions of a certain fraction of nitrocellulose (Fraction No. 1). The data are obtained by the dynamic light scattering method.

Fractionation of the initial sample of colloxylin from the water-acetone solution gives satisfactory results. According to the data given, the distributions are almost monomodal. Characteristic distribution histograms obtained by the dynamic light scattering method for solutions with different concentrations of the same nitrocellulose fraction are shown in Figure 2. More scattering intensity distribution histograms vs. the hydrodynamic diameter for different concentrations solutions of a certain fraction of nitrocellulose (Fraction No. 2–6) are shown in Figures S1–S5 (Supplementary Materials).

**Figure 3:**
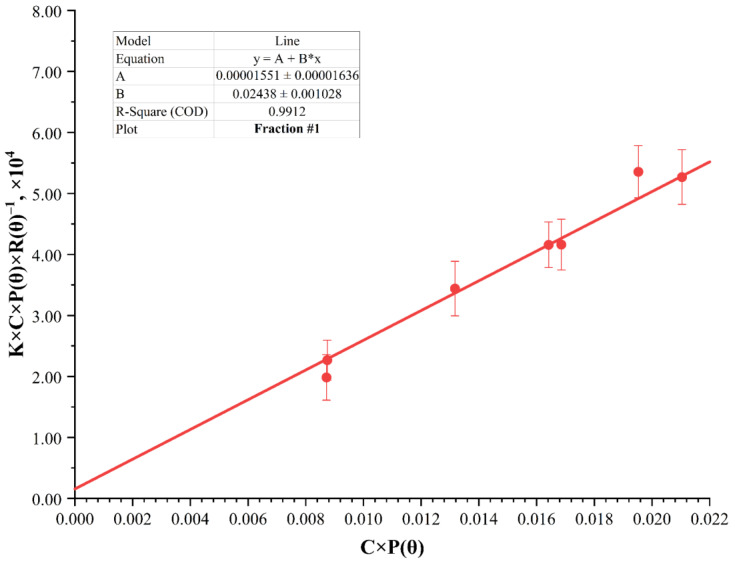
Debye–Zimm diagram in *K·C·P(θ)·R(θ)*^−1^ and *C·P(θ)* coordinates for nitrocellulose fraction No. 1.

In the classical Debye–Zimm method, the correction *P(θ)* is introduced if there is no possibility of measuring at different angles and constructing a Zimm diagram. Then the dependence *K·C·R(θ)*^−1^ *= f(C)* is approximated to the zero concentration value to determine the (*M_W_*·*P(θ)*)^−1^ parameter. However, it can be seen from the distribution histograms that both the hydrodynamic diameter and the scattering intensity are different for each solution. The feature of our proposed method is to the use of special coordinates *K·C·P(θ)·R(θ)*^−1^ *= f(C·P(θ))*. This allows using the different corrections *P(θ)* for each individual concentration. The calculation *P(θ)* based on hydrodynamic radius of cellulose nitrate in solution. This allows us to consider some characteristics of the polymer behavior in solution, depending on its concentration. To process the data obtained via the dynamic light scattering method, the parameters of the Rayleigh–Zimm equation *P(θ)*, *R(θ)*, *K*, and their products *C·P(θ)* and *K·C·P(θ)·R(θ)*^−1^ were calculated for solutions with different concentrations of each nitrocellulose fraction (Table 1). Approximation to the zero value of *C·P(θ)* allows obtaining the value of the (*M_W_*)^−1^ parameter (Figure 3).

The values of the *P(θ)*, *R(θ)*, and *K* parameters and their products *C·P(θ)* and *K·C·P(θ)·R(θ)*^−1^ of the first nitrocellulose fraction are given in Table 1. The values of the refractive index increment for different fractions of nitrocellulose were equal.

After having approximated these parameters using the Debye–Zimm method, the mass average molar mass value of the nitrocellulose fraction No. 1 was found to be equal to 64.5 ± 17.0 kDa. For the fractions No. 2, No. 3, No. 4, No. 5, and No. 6, these values were equal to 37.0 ± 8.6, 29.4 ± 9.9, 22.2 ± 5.6, 18.8 ± 6.3, and 7.98 ± 3.13 kDa, respectively.

**Figure 4:**
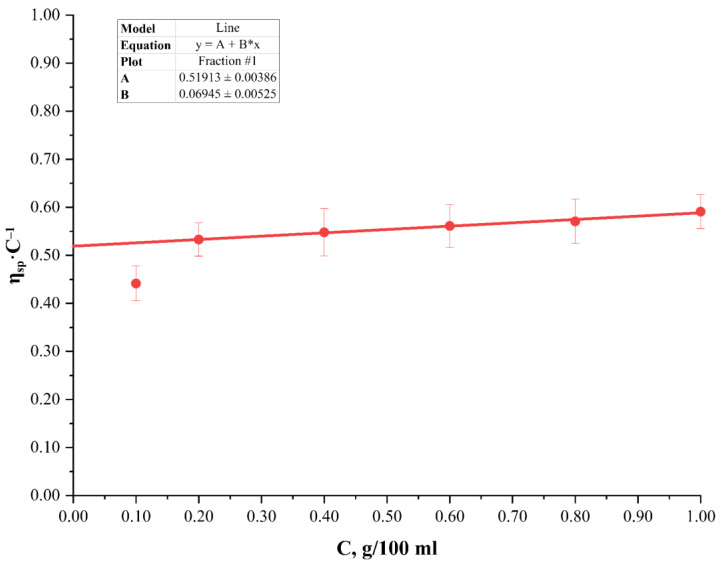
Dependence of the specific viscosity *η_sp_* on the nitrocellulose mass concentration C (fraction No. 1).

Viscometric determination of the polymerization degree is most often used in practice as it is much simpler to perform and does not require expensive equipment. However, the viscometric determination requires considering the nitrogen content (nitration degree) and gives an average integral result of the polymerization degree and molar mass. It is impossible to obtain the molar mass distribution by this method. However, given that this method is simple and thus widely used, it is reasonable to compare the data obtained by the dynamic light scattering and viscometry methods. To achieve this, the flow time through a capillary viscometer of nitrocellulose fraction solutions with different concentrations was measured. Observed relative viscosity values were calculated based on the obtained data. By extrapolating the dependence of the specific viscosity *η_sp_* on the mass concentration C to the infinitely dilute solution, the values of intrinsic viscosity [*η*]*_sp_* were found (Figure 4). More dependences of the specific viscosity *η_sp_* on the nitrocellulose mass concentration C for different concentrations solutions of a nitrocellulose fraction (fraction No. 2–6) are shown in Figure S6, Figure S7, Figure S8, Figure S9 and Figure S10 (Supplementary Materials). The intrinsic viscosity of the studied nitrocellulose solution with a nitrogen content *ω(N)* is reduced to the corrected value of the nitrocellulose solution intrinsic viscosity [*η*]_13.6_ with a nitrogen content of 13.6%, which is taken as standard, by multiplying it by the conversion factor *R_ω(N)_*^13.6^. The average degree of polymerization is calculated using the Newman–Loeb–Conrad formula with the corrected value of the intrinsic viscosity.

Reverse potentiometric titration of nitrocellulose with Fe(II) solution after treatment with sulfuric acid shows a nitrogen mass content in the range from 8.37 to 9.87% by weight. The values of the [*η*]*_sp_*, *R_ω(N)_*^13.6^, [*η*]_13.6_ parameters, and viscosity average degree of polymerization for different nitrocellulose fractions are given in Table 2. When using the molecular formula of nitrocellulose, it is recommended to choose a four-fold cellulose formula, rather than a one-fold one [63]. This way, depending on the number of introduced nitrogroups, the mass average molar mass of the nitrocellulose monomer unit changes (Table 3).

The nitrogen content in the obtained fractions, ranging from 8.37 to 9.87% by weight, allows us to correlate and calculate the mass average molar mass of the nitrocellulose monomer unit for each specific fraction of colloxylin according to the following formula of a second-degree polynomial:

$$\bar{M}=0.4742\cdot \omega^{2}\left(N\right)+1.7954\cdot \omega \left(N\right)+173.5$$

Viscosity average degree of polymerization and molar masses are given in Table 4.

In Table 4, the values of the average viscosity molar mass obtained by the viscometric method and the values of the mass average molar mass obtained by the adapted dynamic light scattering method are compared. For fractionated nitrocellulose, these values are observed to be very close to each other. However, for the unfractionated polymer, the data do not match. For the initial polymodal colloxylin sample, the viscosimetry method provides the value of viscosity average molar mass equal to 56.7 ± 5.8 kDa, but the dynamic light scattering method allows us to determine parameters of the three main fractions of colloxylin (Figure 1)—87.3 ± 14.1, 28.3 ± 7.3, and 0.54 ± 0.17 kDa. The reason for this lies in the inability to obtain molar mass distribution by the viscosimetry method. We have attempted to obtain data on the molar mass distribution of a colloxylin sample by means of size exclusion chromatography. This method allows obtaining a true molar mass distribution. However, colloxylins with a relatively low nitration degree are extremely poorly soluble in solvents typically used for size exclusion chromatography, such as N,N-dimethylformamide or tetrahydrofuran. Chromatographic column manufacturers strongly recommend not changing the solvent. Unfractionated colloxylin samples are only partially resolved in these solvents. The dissolved part of the sample in tetrahydrofuran was examined by size exclusion chromatography and molar mass values were obtained: *M_N_* = 227 Da, *M_W_* = 608 Da, PDI = 2.67 (Figure S11, Supplementary Materials). Obviously, the results are reduced due to partial dissolution and are not characteristic of the whole sample.

## 4. Conclusions

A comparison of the values of the viscosity average molar mass and the values of the mass average molar mass obtained by the dynamic light scattering method allows us to conclude that there is a correlation only for narrow monomodal fractions of the polymer. This is important for the engineering and manufacturing fields, where viscometric data on the polymerization degree and mass average molar mass are typically used: the production of nitrocellulose paint and varnishes, the paper industry, the production of films, etc. However, the data differ for the polymodal distribution and the use of the viscometric method is not objectively justified because it gives only a single value of the viscosity-average molar mass, regardless of the number of fractions in the polymer. On the contrary the dynamic light scattering method possesses advantages when analyzing samples with polymodal distributions. Using this method, it is possible to analyze separately the weight average molar masses of each mode in the resulting distribution without physically separating fractions from each other. The proposed modification of the Debye–Zimm method with the construction of the *K·C·P(θ)·R(θ)*^−1^ = *f(C·P(θ))* dependence allows us to consider the characteristics of the polymer behavior in solution depending on its concentration. Proper corrections *P(θ)* for each individual concentration are calculated from its hydrodynamic radius obtained by the dynamic light scattering method. The dynamic light scattering method is shown to have an advantage over SEC due to the possibility of replacing the solvent with a more suitable one.

## Figures and Tables

**Table 1:** The values of the *P(θ)*, *R(θ)*, and *K* parameters and their products *C·P(θ)* and *K·C·P(θ)·R(θ)*^−1^ obtained by the dynamic light scattering method for solutions with different concentrations of a certain fraction of nitrocellulose (Fraction No. 1).

C, g/100 mL	I(X), %	D, nm	*P(θ)*	*R(θ*)	*K*	*C·P(θ)*	*K·C·P(θ)·R(θ)*^−1^ × 10^4^
0.0100	2.879	1425	2.104	85.26	2.135	0.02104	5.27 ± 0.45
0.00900	2.920	1267	1.873	86.48	2.135	0.01686	4.16 ± 0.42
0.00800	2.760	1090	1.646	81.74	2.135	0.01317	3.44 ± 0.45
0.00700	2.847	1573	2.345	84.31	2.135	0.01642	4.16 ± 0.37
0.00600	2.628	2036	3.254	77.84	2.135	0.01952	5.35 ± 0.43
0.00500	2.781	1174	1.749	82.36	2.135	0.008745	2.27 ± 0.33
0.00400	3.958	1473	2.179	117.2	2.135	0.008717	1.98 ± 0.37

**Table 2:** The values of the nitrogen mass content in different fractions of colloxylin and other parameters of the viscometric determination of the polymerization degree and the colloxylin molar mass.

Fraction	Nitrogen Content in CellNO_3_, % by Weight	Specific Viscosity [*η*]*_sp_*	Coefficient *R_ω(N)_*^13.6^	Corrected Intrinsic Viscosity [*η*]_13.6_	Degree of Polymerization *P_η_*
1	8.39 ± 0.46	0.5191	5.090	2.643	264
2	9.60 ± 0.55	0.3936	3.513	1.383	138
3	9.68 ± 0.58	0.3462	3.426	1.186	119
4	9.27 ± 0.44	0.1903	3.881	0.7384	73.8
5	9.66 ± 0.40	0.1796	3.448	0.6195	61.9
6	9.87 ± 0.47	0.09024	3.227	0.2912	29.1

**Table 3:** Modified formulas of nitrocellulose molecular and molar mass of monomer units depending on the nitrogen content.

Molecular Formula of Nitrocellulose	Molar Mass of CellNO_3_, Da	Nitrogen Content in CellNO_3_, % by Weight	Mass Average Molar Mass of CellNO_3_ Monomer Unit, Da
C_24_H_28_O_8_(NO_3_)_12_	1188.5	14.14	297.1
C_24_H_29_O_9_(NO_3_)_11_	1143.5	13.47	285.9
C_24_H_30_O_10_(NO_3_)_10_	1098.5	12.75	274.6
C_24_H_31_O_11_(NO_3_)_9_	1053.5	11.97	263.4
C_24_H_32_O_12_(NO_3_)_8_	1008.5	11.11	252.1
C_24_H_33_O_13_(NO_3_)_7_	963.5	10.18	240.9
C_24_H_34_O_14_(NO_3_)_6_	918.5	9.15	229.6
C_24_H_35_O_15_(NO_3_)_5_	873.5	8.02	218.4
C_24_H_36_O_16_(NO_3_)_4_	828.6	6.76	207.1
C_24_H_37_O_17_(NO_3_)_3_	783.6	5.36	195.9
C_24_H_38_O_18_(NO_3_)_2_	738.6	3.79	184.6

**Table 4:** The values of different parameters of colloxylin viscosity average molar mass obtained via viscosimetry and mass average molar mass obtained by direct light scattering.

Fraction	Nitrogen Content in CellNO_3_, % by Weight	Viscosity-Average Degree of Polymerization *P_η_*	Mass Average Molar Mass of a CellNO_3_ Molecular Unit, Da	Viscosity-Average Molar Mass *M_η_*, kDa	Mass Average Molar Mass Determined by the Dynamic Light Scattering Method, *M_W_*, kDa
1	8.39 ± 0.46	264	222.0	58.7 ± 11.3	64.5 ± 17.0
2	9.60 ± 0.55	138	234.4	32.4 ± 6.1	37.0 ± 8.6
3	9.68 ± 0.58	119	235.3	27.9 ± 4.7	29.4 ± 9.9
4	9.27 ± 0.44	73.8	230.9	17.1 ± 1.8	22.2 ± 5.6
5	9.66 ± 0.40	61.9	235.1	14.6 ± 2.2	18.8 ± 6.3
6	9.87 ± 0.47	29.1	237.4	6.92 ± 1.10	7.98 ± 3.13

## Data Availability

Not applicable.

## Supplementary Materials

The following supporting information can be downloaded at: https://www.mdpi.com/article/10.3390/polym15020263/s1, Figure S1. Scattering intensity distribution histograms vs. the hydrodynamic diameter for different concentrations solutions of a certain fraction of nitrocellulose (Fraction No. 2). The data are obtained by the dynamic light scattering method; Figure S2. Scattering intensity distribution histograms vs. the hydrodynamic diameter for different concentrations solutions of a certain fraction of nitrocellulose (Fraction No. 3). The data are obtained by the dynamic light scattering method.; Figure S3. Scattering intensity distribution histograms vs. the hydrodynamic diameter for different concentrations solutions of a certain fraction of nitrocellulose (Fraction No. 4). The data are obtained by the dynamic light scattering method; Figure S4. Scattering intensity distribution histograms vs. the hydrodynamic diameter for different concentrations solutions of a certain fraction of nitrocellulose (Fraction No. 5). The data are obtained by the dynamic light scattering method; Figure S5. Scattering intensity distribution histograms vs. the hydrodynamic diameter for different concentrations solutions of a certain fraction of nitrocellulose (Fraction No. 6). The data are obtained by the dynamic light scattering method; Figure S6. Dependence of specific viscosity *η_sp_* on nitrocellulose mass concentration C (Fraction No. 2); Figure S7. Dependence of specific viscosity *η_sp_* on nitrocellulose mass concentration C (fraction No. 3); Figure S8. Dependence of specific viscosity *η_sp_* on nitrocellulose mass concentration C (fraction No. 4).; Figure S9. Dependence of specific viscosity *η_sp_* on nitrocellulose mass concentration C (fraction No. 5); Figure S10. Dependence of specific viscosity *η_sp_* on nitrocellulose mass concentration C (fraction No. 6); Figure S11. Size exclusion chromatography curves (black line) normalized to unit area of unfractionated colloxylin and deconvolution of data by Gaussian fitting (color curves).
