# Supplementary material for: Special Aspects of Nitrocellulose Molar Mass Determination by Dynamic Light Scattering
Source: Polymers (Basel). 2023 Jan 4;15(2):263. doi: 10.3390/polym15020263 (PMC9862043; doi:10.3390/polym15020263)
Supplement: Supplementary file 1 [file polymers-15-00263-s001.zip › polymers-2077060-supplementary.pdf]

polymers

MDPI

Supplementary Materials

Special aspects of nitrocellulose molar mass determination by dynamic light scattering

Roman Solovov*, Anfisa Kazberova and Boris Ershov

Frumkin Institute of Physical Chemistry and Electrochemistry of the Russian Academy of Sciences, 40 Obruchev Street, Moscow, 117342 Russia; perevoznikova1723@mail.ru (A.K.); ershov@ipc.rssi.ru (B.E.)
* Correspondence: roman_solovov@mail.ru

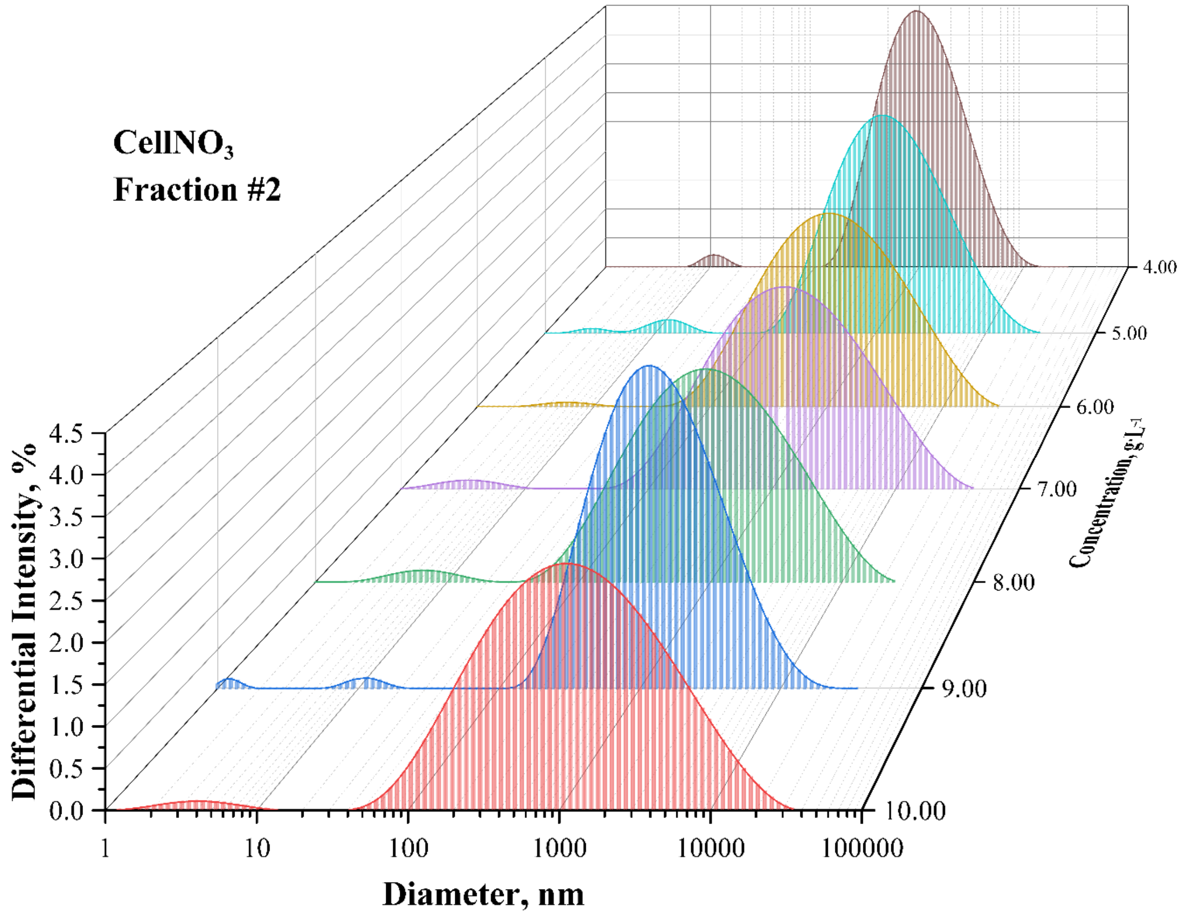

Figure S1. Scattering intensity distribution histograms vs the hydrodynamic diameter for different concentrations solutions of a certain fraction of nitrocellulose (Fraction №2). The data is obtained by the dynamic light scattering method.

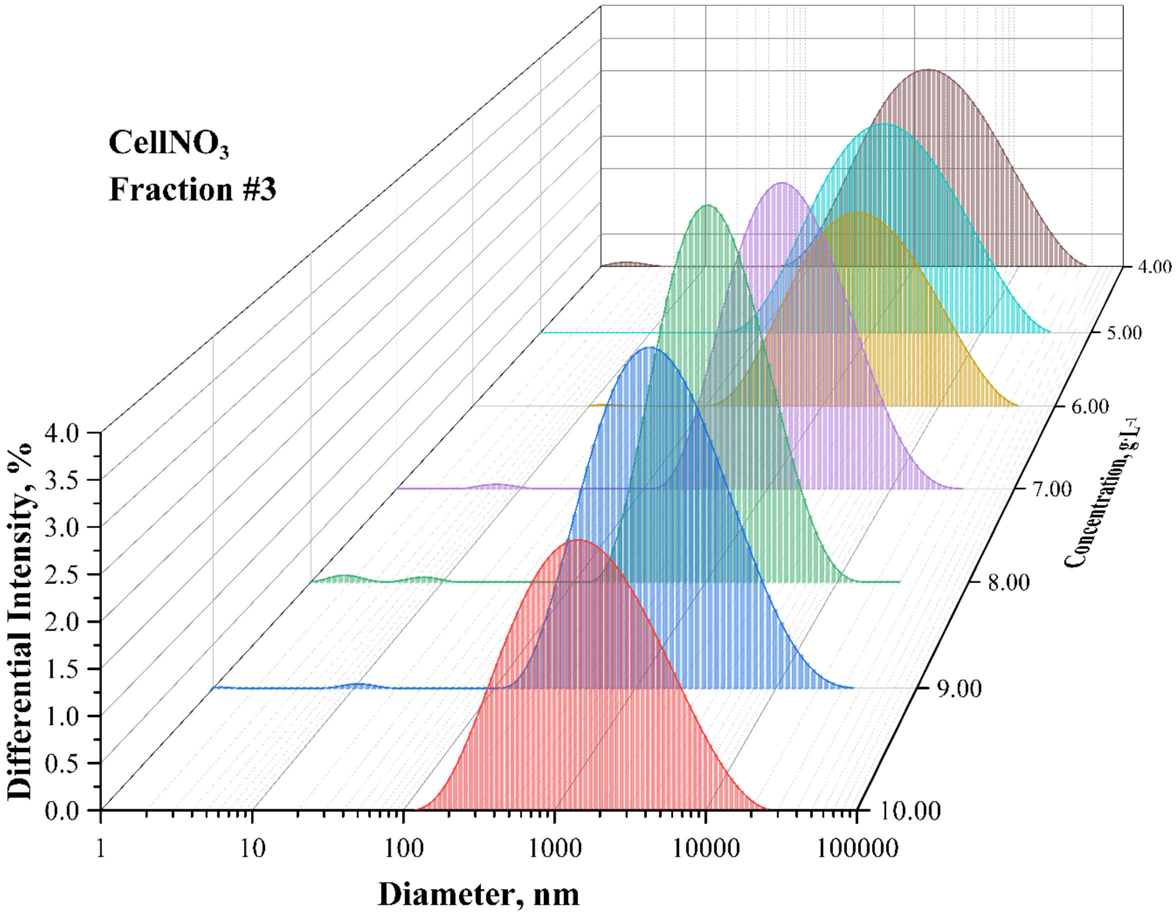

Figure S2. Scattering intensity distribution histograms vs the hydrodynamic diameter for different concentrations solutions of a certain fraction of nitrocellulose (Fraction №3). The data is obtained by the dynamic light scattering method.

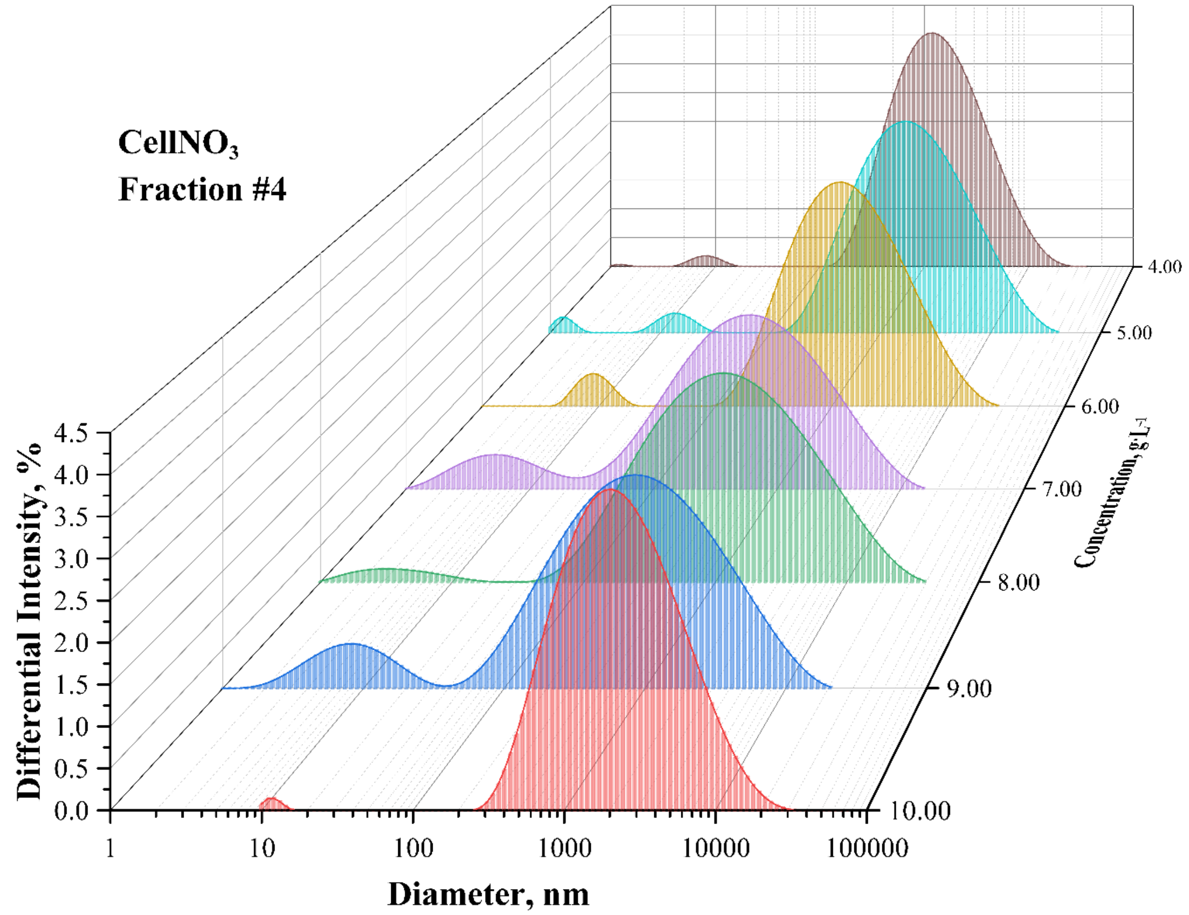

Figure S3. Scattering intensity distribution histograms vs the hydrodynamic diameter for different concentrations solutions of a certain fraction of nitrocellulose (Fraction №4). The data is obtained by the dynamic light scattering method.

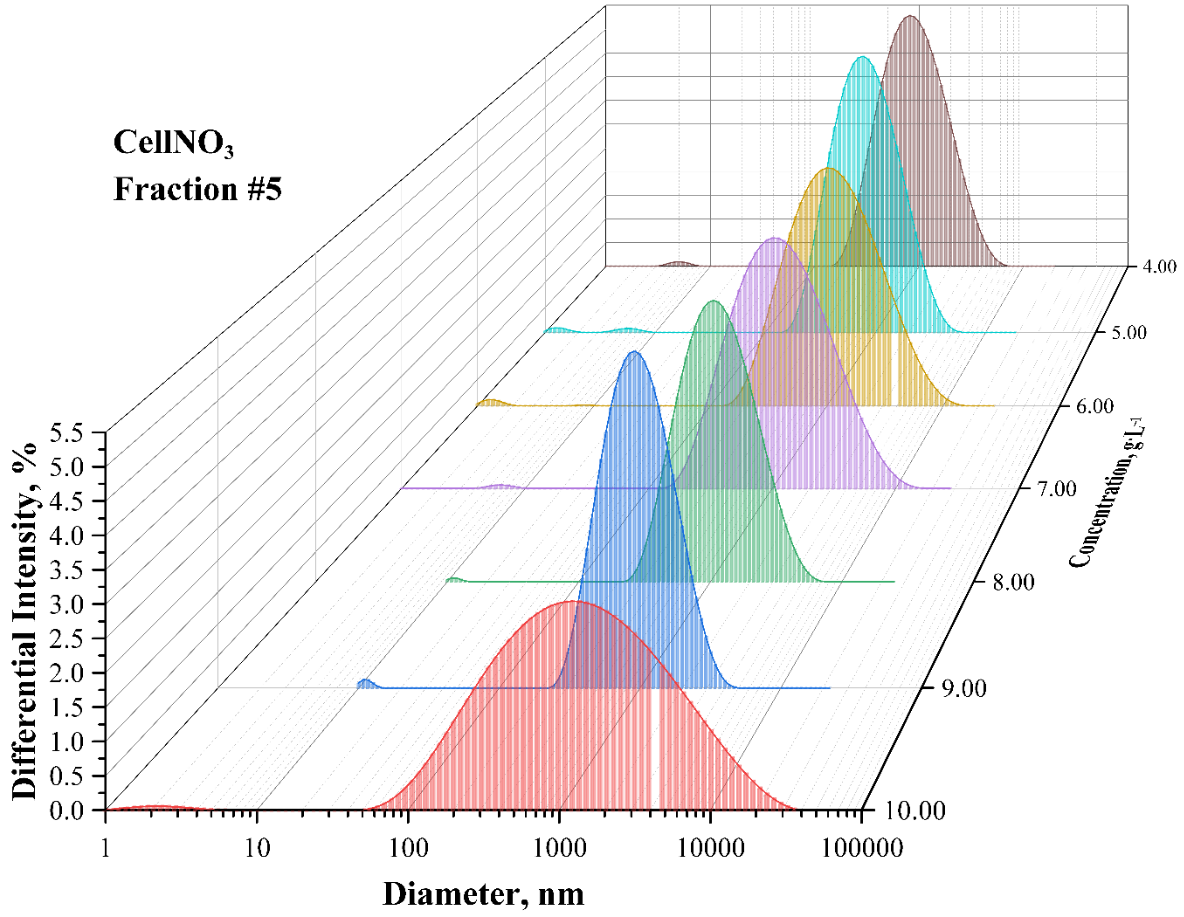

Figure S4. Scattering intensity distribution histograms vs the hydrodynamic diameter for different concentrations solutions of a certain fraction of nitrocellulose (Fraction №5). The data is obtained by the dynamic light scattering method.

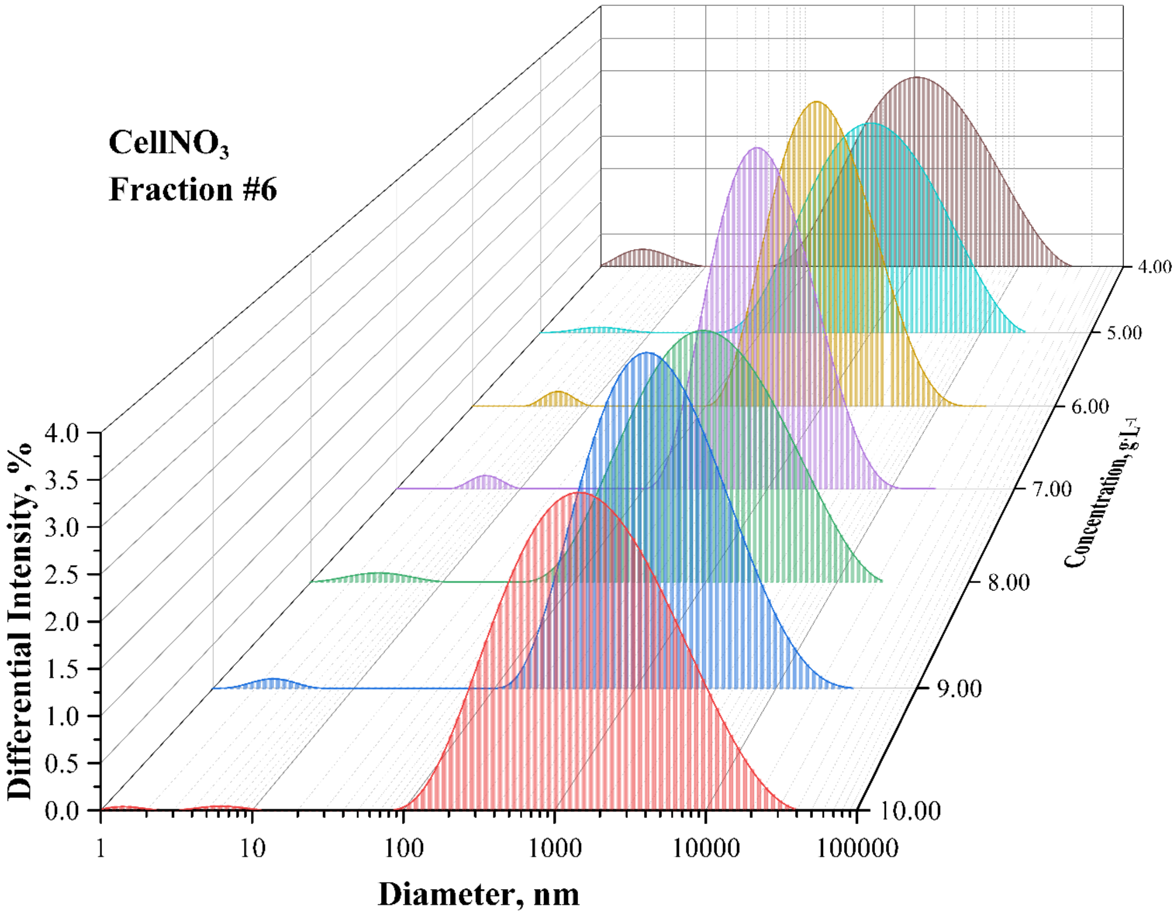

Figure S5. Scattering intensity distribution histograms vs the hydrodynamic diameter for different concentrations solutions of a certain fraction of nitrocellulose (Fraction №6). The data is obtained by the dynamic light scattering method.

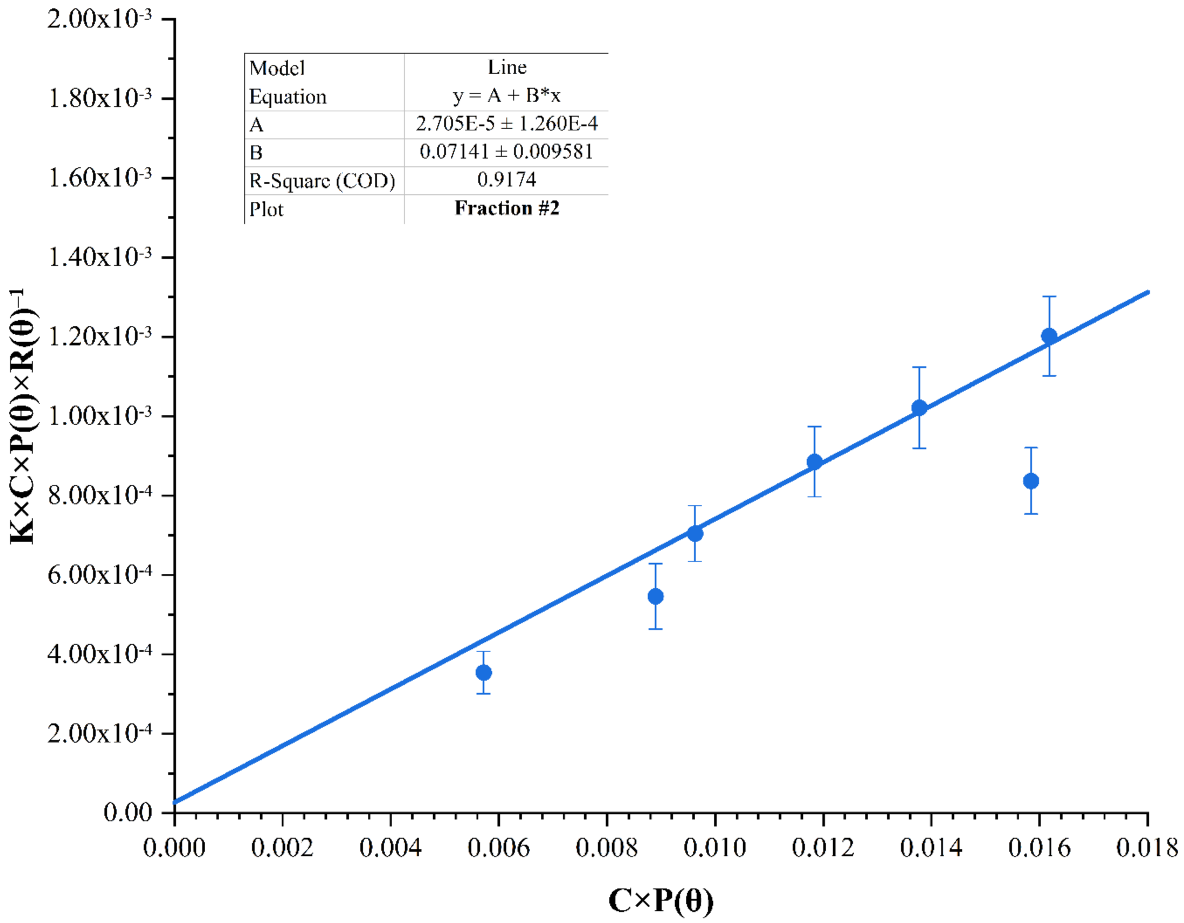

Figure S6. Dependence of specific viscosity η_{sp} on nitrocellulose mass concentration C (fraction №2).

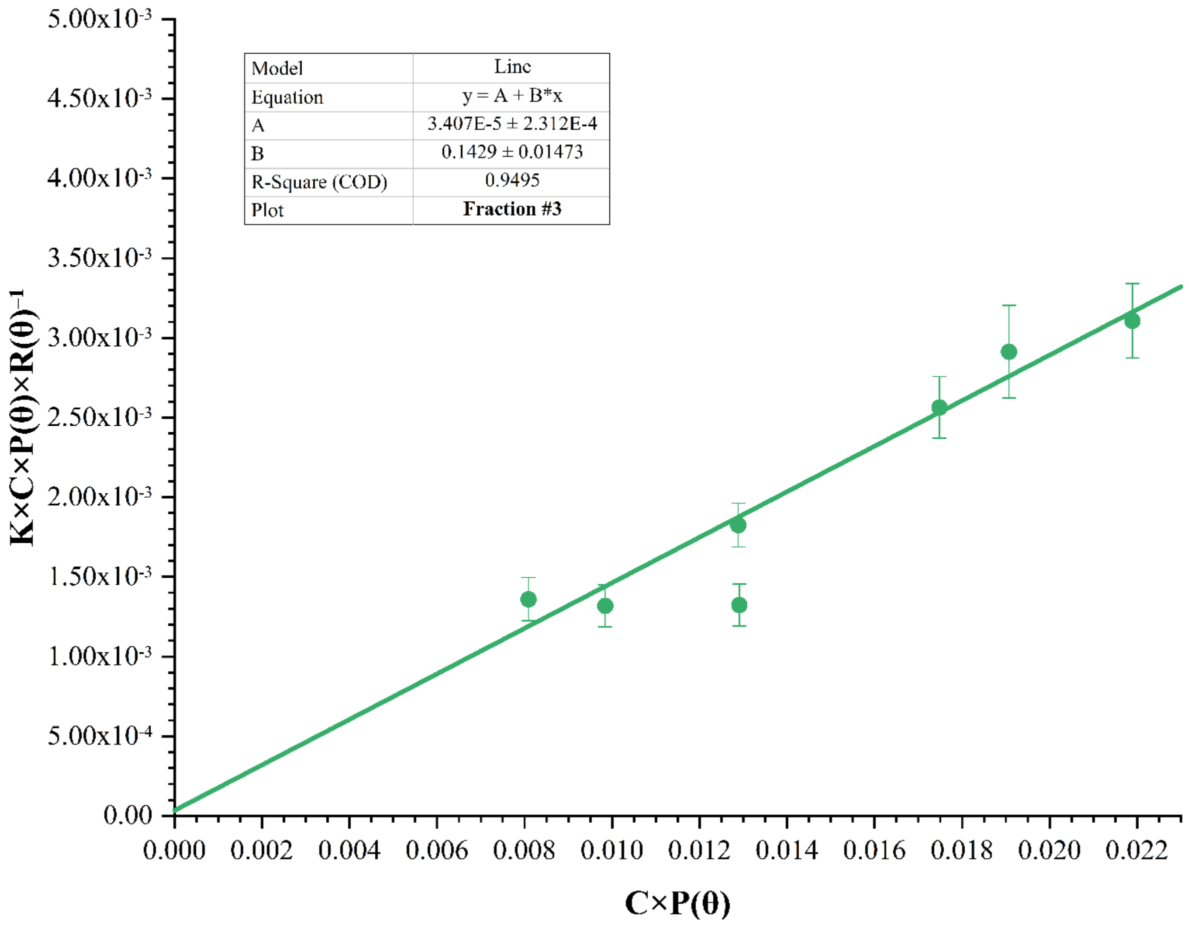

Figure S7. Dependence of specific viscosity η_{sp} on nitrocellulose mass concentration C (fraction №3).

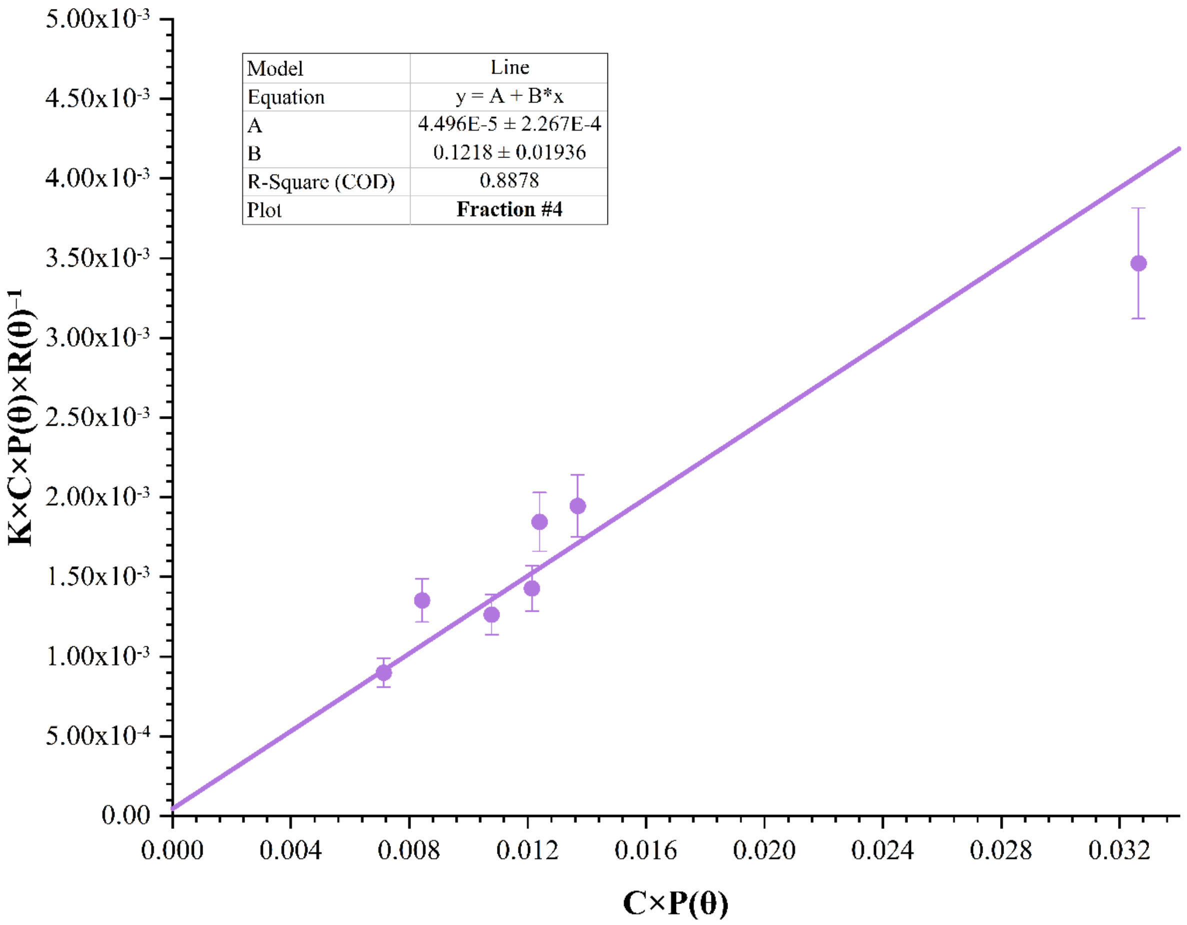

Figure S8. Dependence of specific viscosity η_{sp} on nitrocellulose mass concentration C (fraction №4).

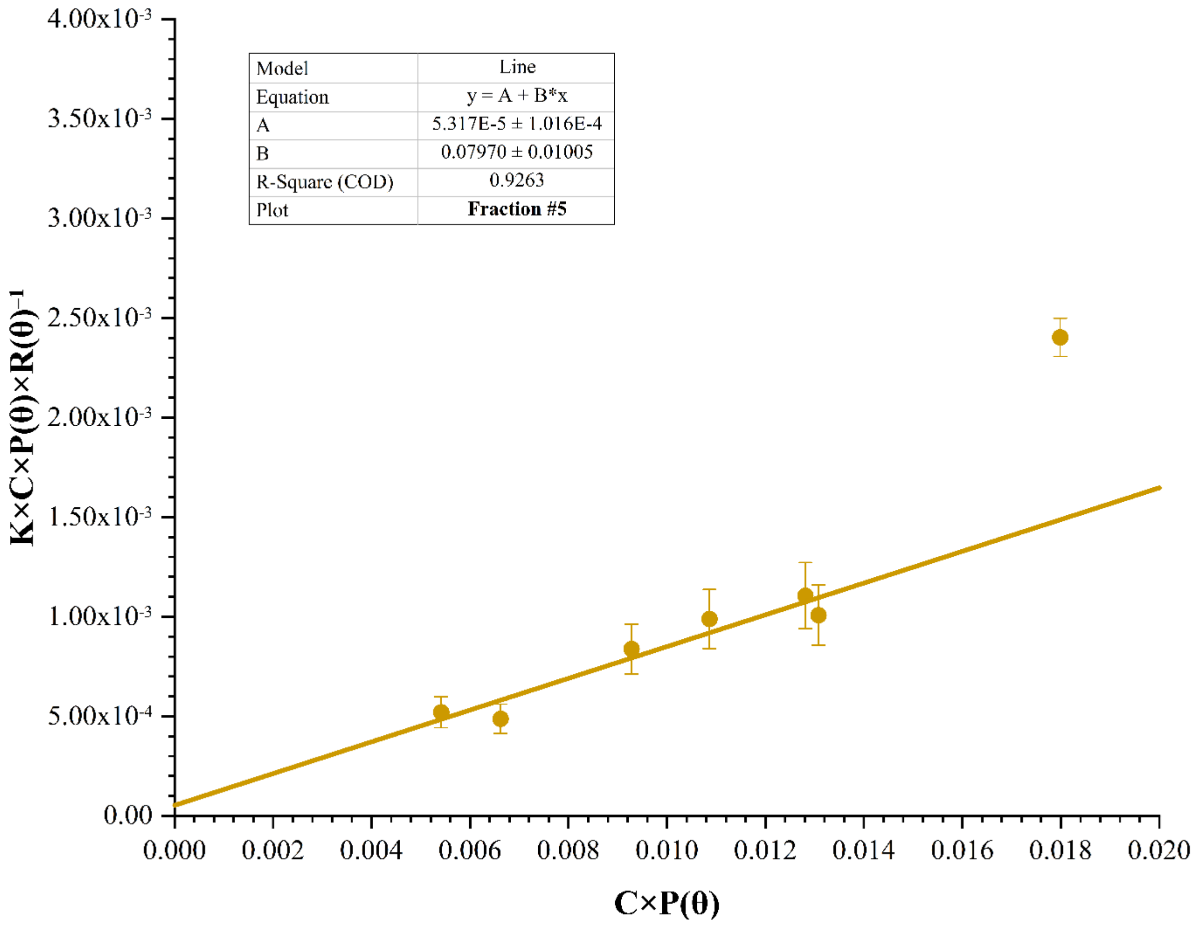

Figure S9. Dependence of specific viscosity η_{sp} on nitrocellulose mass concentration C (fraction №5).

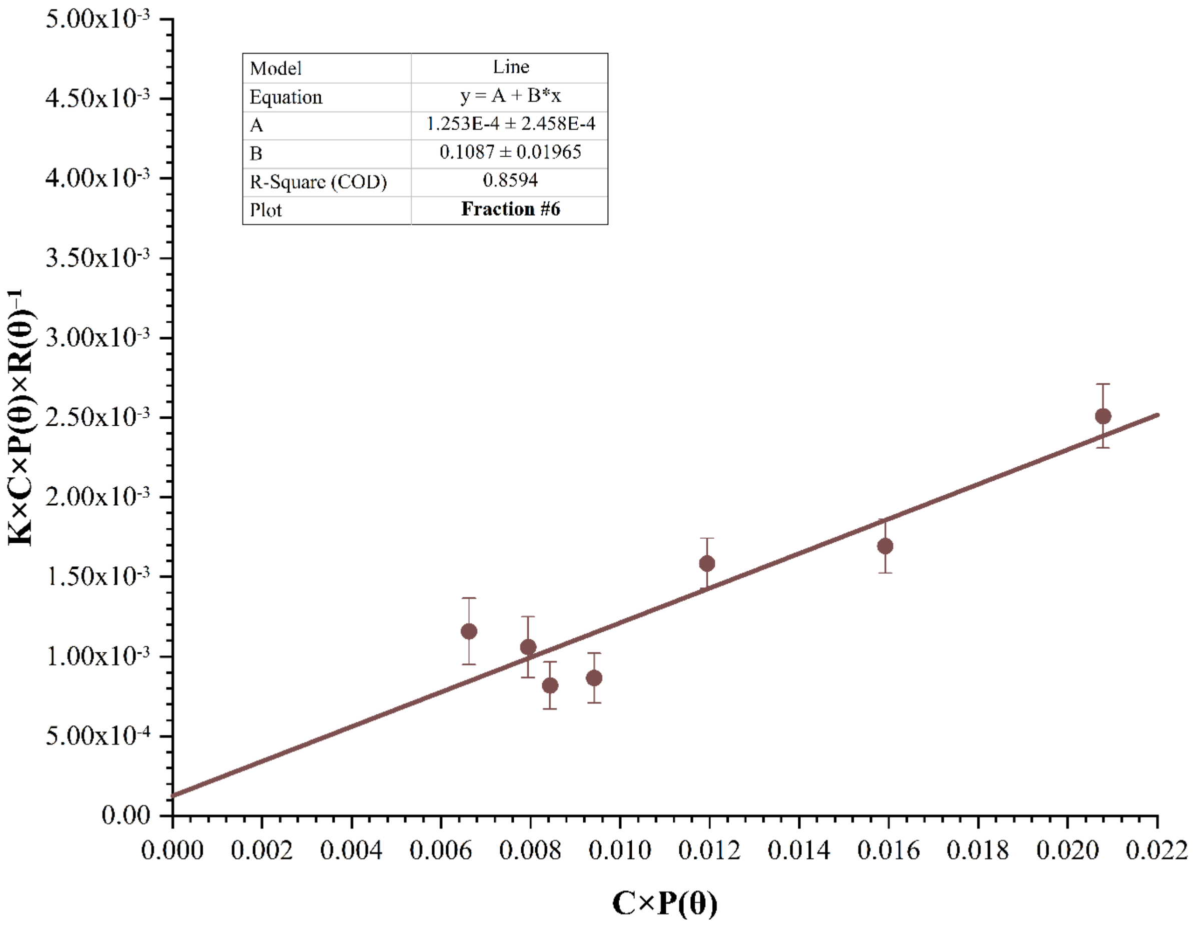

Figure S10. Dependence of specific viscosity η_{sp} on nitrocellulose mass concentration C (fraction №6).

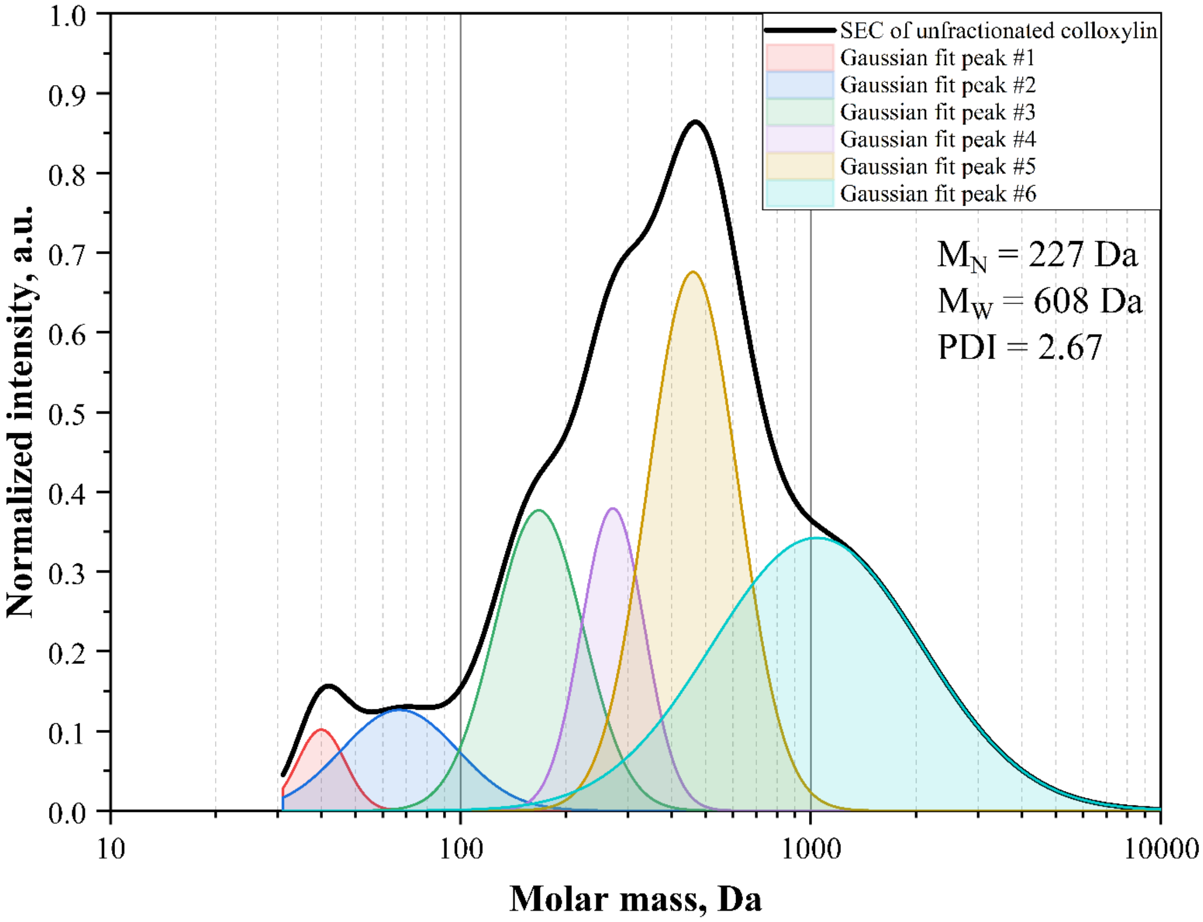

Figure S11. Size exclusion chromatography curves (black line) normalized to unit area of unfractionated colloxylin and deconvolution of data by Gaussian fitting (color curves).
